# On Exostoses: Their Character

**Published:** 1848-10

**Authors:** 


*On Exostoses: their Character.*—A valuable memoir has been published in the *Revue Médico Chirurgicale,* illustrated by numerous cases, on Exostoses, and on the operations necessary to their removal, by M. Roux. We here give the conclusions at which the author arrives :

1. Among the different tumors met with on bones, some of them only are to be termed exostoses, and from them all others which have borne the name should be carefully distinguished.

2. True exostoses are constituted, like the bones themselves, chiefly of the spongy or cancellated variety, and only covered by a thin lamina of the compact bony tissue; or, on the contrary, they may be entirely composed of compact tissue, and then their consistence is very hard and resembles ivory.

3. Every exostosis having either of the preceding characters, is attached to the bone from which it is developed by a constricted base, forming a pedicle, short and thick, proportionate to the volume of the tumor.

4. After a certain time, exostoses cease to grow, and never pass beyond a certain degree of development. This definite volume is proportionate to that of the bone on which they may be implanted, being small on small bones, of large magnitude on great ones.

5. They are mostly merely contiguous to the surrounding soft parts: sometimes, however, they contract adhesions with them.

6. Exostoses do not degenerate, but tend indefinitely to preserve their primitive structure, or, at least, their tissue may acquire, by age, a greater density, as does that of the bones, when they are formed in young subjects.

7. According to the situation they occupy, they may constitute a deformity only; or they may produce pain and difficulty to the functions of the surrounding organs; or, lastly, they may, in some cases, act as an obstacle to the performance of their functions.

8. If their position do not render them inaccessible, they may almost always be removed; and their removal in nearly every case is indicated, either to do away with a great deformity, or constant suffering and uneasiness, or to re-establish the functions of an organ in their full and regular play.

9. The removal of exostoses may, in the greater number of cases, be proceeded with, without previously exposing the tumor, and without having great difficulties to surmount.

10. The operation may be followed by serious consequences, either because of the peculiarity of the locality of the tumor, or on account of its relations and connections with neighboring organs, or from a vitiated state of the constitution in the individual operated on. Nevertheless, the removal of exostoses is generally successful.

